# A Perspective on The Göktaş Definition of Family Medicine/General Practice: A Review of Singapore Quality

**DOI:** 10.12669/pjms.41.4.11382

**Published:** 2025-04

**Authors:** Olgun Goktas

**Affiliations:** 1 Olgun Goktas, Associate Professor, Uludag University Family Health Center, Nilufer, Bursa, Turkey

**Keywords:** Family medicine, General practice, Göktaş, definition, Quality, Singapore

## Abstract

Family medicine/general practice is a medical field that provides health services within the health systems of countries, and it is affected by the development level and quality of the countries. The Göktaş Definition of Family Medicine/General Practice, which summarizes family medicine in the history of medicine and humanity, forces new perspectives from the past to the future. Countries have focused on family medicine and have been intensive in efforts to improve their health systems for years. With its complex content, family medicine is affected by the quality of health systems on the one hand and determines the health quality of countries on the other. Therefore, the development of family medicine is more possible in a country with a human development level. This narrative review was planned to evaluate the strengths and weaknesses of countries and centers that can serve as examples of family medicine in all aspects of the literature. For this purpose, a literature review was conducted in October 2024 from Google Scholar, PubMed, Web of Science, SCOPUS search engines and databases. Two hundred twenty five articles and reports were examined, and 54 English articles, reports, and online and textbook sections that were appropriate to the subject’s essence were taken as source references. This review investigates the current status, possibilities, limitations, and future perspectives of Singapore family medicine and health system in the context of the Göktaş definition.

## INTRODUCTION

Health services and outcomes vary across countries depending on the family medicine practice they have. These differences lead to a lack of vision worldwide and deviations from the general characteristics of the family medicine discipline, especially its core competencies. The acceptance of family medicine as a well-practiced and quality service in a country may vary from country to country. These differences may inevitably raise the question of which form of family medicine practice is best.

Family medicine practices in developed countries can be given as an example. However, when examining the family medicine practices of large countries in terms of population and geography, general problems can hide some characteristics. On the other hand, examining the health system and family medicine practices in a small developed country in terms of population and geography may be useful in terms of evaluating all dimensions. In this review, Singapore’s health system, family medicine, and the factors affecting it are evaluated in the context of Göktaş’s definition of family medicine.

Medicine has such an influence on people that it cannot remain indifferent to the fact that none of the sick people receive the best treatment. The National Health Act of the United Kingdom, with the dedication of many doctors, expanded this idea and finally led to its adoption by the State. The United Nations Organization also declares that man should be freed from the fear of disease.^1^

By definition, the family doctor’s different approach to individuals in healthcare systems is important and always necessary.^2^,^3^ The Göktaş definition of Family Medicine/General Practice^4^ within the scope of the family doctor’s holistic relationship with the individual and the patient, well defines family medicine and all the characteristics of the family doctor. The future and sustainability of family medicine are thanks to individual and patient-focused approaches and require family medicine practices with quality values determined by measurable standards.^5^-^10^

Due to its complex content, family medicine is affected by the quality of the country’s health system. In addition, family medicine determines the quality of the country’s health services and outcomes. The development and sustainability of family medicine are more possible in a country with high quality in all aspects and especially in a country with a high level of human development. The United Nations Development Program (UNDP) ranks countries according to their Human Development Indexes (HDI)^11^ in its annual Human Development Report.^12^ This report states that increasing inequality and political polarization create a deadlock between countries. This situation makes it difficult to follow the goals of the 2030 Agenda for Sustainable Development^13^, and the Paris Agreement.^14^ Therefore, even in a rich, large, and developed country, inequalities and political polarization make it difficult to improve health.^15^,^16^ The Astana Declaration, which was held in 2018 with the participation of heads of state and government representatives, aimed to strengthen individuals and societies and to organize a safe, comprehensive, accessible, and high-quality system, especially in primary healthcare services.^17^

Singapore has a special place among developed countries. According to the annual Human Development Report of the United Nations Development Program (UNDP), Singapore ranks 9th in terms of countries’ Human Development Index (HDI). Singapore is a very small country and an island surrounded by the sea. These characteristics may have allowed it to design and implement quality family medicine and improve the quality of family medicine.

In this study, Singapore’s health system and health level in terms of family medicine are analyzed from the perspective of the Göktaş Definition of Family Medicine/General Practice to update, modify, and raise the standards of family medicine to reach a global example.

### Singapore:

Singapore is a metropolitan island country with a special history^18^, located at the southern tip of the Malay Peninsula in Southeast Asia, with an area of 710 square kilometers and a population of approximately five million people, comprising four major ethnic groups. It has been governed by a parliamentary democracy since its independence in 1965. Singapore has a rich diversity of cultures and ethnicities, with world-class infrastructure, and an island-wide transportation network.^19^ It has made significant political developments in its short history.^20^ In addition to its young population, the city-state is rapidly shifting towards an increasingly elderly society.^21^

### Singapore health system and family medicine/general practice:

In Singapore, the concept of medical professionalism has been legislated by the Singapore Medical Council (SMC) Ethical Code and Ethical Guidelines, which were issued in 2016 and are constantly updated.^22^,^23^

After Singapore gained independence in 1965, it developed new legislation based on personal responsibility and improved its healthcare system. New reforms were introduced in 2012 and three key fundamental changes were introduced in 2016: from healthcare to health, from hospital to community, and from quality to value.^24^

Before implementing new models in the health system, it is recommended that issues such as human-centeredness, innovation and integration be scaled and followed in new pilot models to be created.^25^,^26^ The Singapore government has launched reforms to help pay for medical treatment for the elderly, expand insurance coverage and increase primary care services for those who qualify.^27^

At a conference in 1991, four main points were proposed to make Singapore the leading healthcare center in the region. These recommendations were that Singapore should focus primarily on quality healthcare and professionalism, develop as a benchmark for high quality and standards as a center for healthcare education, become a center for healthcare research and development (R&D), and carry its healthcare brand abroad with its culture and ethics. The Ministry of Health planned to revise public institutions and open them to international services by also developing communication, finance, and information technology.^28^ It was emphasized that Singapore could maintain its current quality in medical and health services by integrating with the Ministry of Health, universities, and all health workers and that this was a vital necessity.^29^

A study in Singapore suggests that family physicians’ camaraderie and knowledge of disease management are important in integrating structures at different levels of health.^30^ Many countries have implemented family medicine in their healthcare systems since the Alma-Ata Declaration of 1978 but without success in quality. While some of these challenges apply to Singapore, family medicine as practiced in Singapore has been key to the long-term success of the Singapore healthcare system.^31^

Singapore has quickly brought the COVID-19 outbreak under control, thanks to the quality of its infrastructure and institutions, especially over the last 10 years.^32^ Multi-sectoral cooperation and widespread sharing of data to improve health services and increase quality in Singapore^33^, and it is stated that to obtain accurate results, a person-centered sustainable health system should be developed and preventive health services should be adopted.^34^

In a study in Singapore, primary care specialists’ low ratings of the system were due to physicians’ low earnings, academic family medicine standards, and workload.^35^ In contrast to these results, the work of primary care physicians^36^-^41^ provided successful results in terms of quality.

## METHODS

This narrative review was planned to evaluate the strengths and weaknesses of countries and centers that can serve as examples of family medicine in all aspects of the literature. For this purpose, a literature review was conducted in October 2024 from Google Scholar, PubMed, Web of Science, SCOPUS search engines and databases. Two hundered twenty five articles and reports were examined, and 54 English articles, reports, and online and textbook sections that were appropriate to the subject’s essence were taken as source references.

### New perspectives for Singapore quality in the context of the Göktas Definition of Family Medicine/General Practice:

The Göktaş definition is a definition that complements the WONCA Europe 2011 family medicine/general practice definition. The WONCA Europe 2011 definition has defined the physical conditions of family medicine in the field of medicine, the characteristics of the clinical discipline, the core competencies, and the duties and responsibilities of the family physician quite well. However, over time, while the family physician works to fulfill his duties and responsibilities under these conditions, he is stuck within the conditions of the health system of the country he is in and with a heavy workload. Over time, the contents of family medicine that need to be updated could not be updated. In addition, the standards that need to be established have become undevelopable within the conditions of the country. Under these conditions, the family physician is forced to work within the legal regulations and heavy workload. Depending on the conditions of the countries, deviations from the basic characteristics of the family medicine discipline increase. This situation has brought to the agenda that the family physician has powers and rights as well as individual duties and responsibilities. At this point, the Göktaş Definition of Family Medicine/General Practice has entered the literature as a definition that ensures that the family physician is given endless authority, protected, and protected due to these difficult and devoted working conditions and that he maintains his agenda.

While providing primary health care services, a family physician must focus on individuals and patients, as well as their families, society, the rules of the health system, the conditions of the office, and the development of their professional knowledge. On the other hand, while working within this busy agenda, they should not be stuck against the risk of legislation. A physician who does not feel comfortable and provides quality service within his busy agenda should not be expected to work like a machine. Therefore, the standards of family medicine must be developed and updated quickly and firmly.

At this point, regions or countries that can be taken as examples of family medicine practices are examined. Even though they are a developed country, there are greater problems in developing family medicine practices in countries with large populations and geographical structures. However, family medicine practices of countries with small populations and geographical lands but high development indices gain value. It is easier to receive feedback reports on the processes of developing, updating, and establishing standards in family medicine in countries with these characteristics. Accordingly, updating and developing the health system and family medicine with alternative approaches will be possible.

### Singapore and its conditions on family medicine/general practice

Can Singapore have quality family medicine practices with its population and land area being smaller than most countries and being a leading country according to development indexes? Even with its land area being mostly an island surrounded by the sea; can it be a pioneer in quality family medicine more appropriately than other countries? Of course, the level of development will affect the quality, but family medicine will continue with the final decisions and practices of a human physician. At this point, it is likely that family medicine in well-educated, protected by the health system, and with more defined standards will be better. The family physician plays the leading role in the health system and family medicine practice. At this point, the family physician needs to be self-confident feel competent, and to do his job motivated. A suitable working environment, good organization, and accessible residential area are essential for the motivation required for the family physician. In medical professionalism, the individual approach to the patient is understood as a commitment to society and its qualities are global.^42^ At this point, the understanding of medical professionalism in family medicine must be at the highest level. These conditions are more likely in developed countries for family physicians and family medicine and increase the quality level. The Göktaş Definition of Family Medicine/General Practice provides a global vision to physicians, people, society, and all other stakeholders of health to reach quality health care thanks to the detailed definition of family physicians and family medicine.

Can the Göktaş Definition of Family Medicine/General Practice be a source of motivation in a country like Singapore that can demonstrate its focus on quality? Can Singapore transfer this quality to neighboring countries? Can Singapore analyze and synthesize family medicine practices from other countries and provide feedback? Contributions and updates that will be obtained through feedback are urgently needed in the medical world and the discipline of family medicine, and these needs are of vital importance.

In a study conducted in Singapore to investigate the problems of improving the quality of family medicine, the general satisfaction of family physicians is remarkable. However, the need to reduce the pressure of lawsuits on medical practice is also emphasized as one of the important solution points.^43^ At this point, family physicians need to be comfortable in the grip of job descriptions, rules, and legislation while performing their duties. The mental state and personal agenda of the family physician are the most valuable assets in their critical duties. Because the family physician is the main player in the health system, and the most critical employee in the business world. The power that the family physician will need while performing their very valuable and critical duty is specified in detail in the Göktaş definition of family medicine. Clinical knowledge, experience, and good practice can be expected from every physician in every profession, but the power that will make the family physician stand out in their much more valuable and human-oriented duty is in the Göktaş definition of family medicine. The mental and personal agenda of the family physician in this definition should be noticed by the rule makers, health institutions as employers, politicians, health organizations, and system managers. The most important point that is essential for quality in family medicine lies in this definition. Singapore’s quality of healthcare and family medicine could benefit from this definition.

In a strategy study conducted in Singapore to transform family medicine into a more robust structure and quality service, it is stated that family medicine plays an important role in the excessive workload. It is emphasized that one of the most important elements for the success of health reform in the implementation of family medicine is to ensure that the general needs of the family doctor are taken into account.^44^ As stated in the Göktaş Definition of Family Medicine/General Practice, the needs of the family doctor are one of the most important elements in the success of the system

Among health services, the management of patients with complex problems that include medical, psychological, social, or behavioral problems is important. The family physician plays an important role in distinguishing these difficult patients, in the follow-up and treatment of patients, in the continuation of consultations, and in the solution of complex health problems that any specialist cannot solve.^45^ The motivational power and personal agenda of the family physician in Göktaş’s definition are key in the solution of complex health problems. The family physician’s success in these problems is one of his most important characteristics and is often not noticed, but this characteristic should be taken into account in sustainable medicine.

A kiosk application where patients can gain most of the information, they need for their complex problems in advance can be useful in reducing unnecessary bureaucracy for individuals applying to their family physician. Reduced bureaucratic burden and health awareness can reduce the unnecessary workload of family physicians and allow them to focus better on the patient.^46^

In contrast to financial centers in a quality health service, the structuring and organization of medical centers and health services is much more complex. Public administration should have an impact on the organization of medical services and it is important to determine the number of doctors to be trained in the training of quality doctors. However, no matter how good the doctors are, the training and quality of support departments and paramedical staff are also important.^47^

In a study in Singapore, it is emphasized that health services in hospitals that are strained by applications due to increasing chronic diseases should be directed to the family physician for community and individual-oriented services. In this study, the complexity of the system, and the reinforcement of the organization for functional integration create difficulties, but the sensitivity of service providers and the loyalty and cooperation of all stakeholders, including physicians, were important.^48^ The quality of health care depends on its good organization and continuity.^49^ Quality in health depends first on the organization of primary health care services, then on its integration with other health institutions and the support of the family physician as in the Göktaş Definition of Family Medicine/General Practice.

Singapore focuses on family medicine and seeks international cooperation by integrating health services, and wants to gradually increase the quality of health organization, education and research, and family medicine practices. These characteristics make Singapore a pioneer country in the region in family medicine and health services.^50^ It develops cooperation with its family medicine clinic and other medical clinics within the National University of Singapore and implements the results in the national health system.^51^ It is important to train high-standard and high-quality family medicine educators. Standardized scientific teaching methods that support the development of family medicine should be developed, along with high-quality teaching experiences.^52^ In the discipline of family medicine, academic structuring, quality field practice, and the presence of a successful scientific journal^53^ are complementary requirements. Collaboration between family medicine and other clinics will of course be reflected in the quality of health services. In such collaborations, family medicine is an indispensable determinant. The Göktaş Definition of Family Medicine/General Practice emphasizes the importance of collaborations between other clinics.

The need for policy, academic structuring, research, integration of health information systems, benefit and safety assessment, and implementation feedback is emphasized for the quality of family medicine and the health system in Singapore.^54^

According to the Göktaş Definition of Family Medicine/General Practice, Singapore has taken important steps throughout its history and is trying to improve the quality of its healthcare system and family medicine. In this context, the Göktaş Definition of Family Medicine/General Practice appreciates Singapore’s ongoing efforts for quality in family medicine and the health system and considers its feedback in the literature.

### Inclusion and exclusion criteria:

This review scanned all literature, especially PubMed, Scopus, and Google Scholar. Among the articles that were suitable for the keywords, articles that were clearer and more applicable, especially those that were suitable for the review, were included in the review. The general characteristics of family medicine and health systems and the physical conditions of Singapore were not mentioned much in the review. On the other hand, articles that included appropriate topics within the scope of quality of Singapore’s family medicine and health system in the context of the Göktaş Definition of Family Medicine/General Practice were examined. Articles that provided general information about family medicine practices and health systems in the literature and articles with similar content and repetitive articles were excluded from the review.

### Limitations:

In the review, only articles that included the quality characteristics of Singapore family medicine practices and the health system in the context of Göktaş Definition of Family Medicine/General Practice were examined and comments were added. Including only articles that could create a perspective on Singapore, not mentioning the family medicine and health systems of other countries, and not comparing with Singapore may create limitations. The characteristics of Singapore were examined by the purpose of the review. More extensive studies are needed to evaluate Singapore’s health system and the economic, political, and cultural factors that may affect the quality of family medicine practice.

## CONCLUSION

Since the scope of family medicine in the health systems of countries is wide, its implementation and results are important. The definition of family medicine, which directly affects the quality of health services and is affected by the systems, should be well known. In this respect, the Göktaş Definition of Family Medicine/General Practice defines all the characteristics of family medicine and family physicians well. In this definition perspective, the future and sustainability of family medicine require that it has a quality value determined by measurable standards.

In this context, Singapore has a special place among developed countries. Unlike other developed countries in terms of population and geography, Singapore is a very small country and its borders are in the form of an island surrounded by the sea. These characteristics may have provided it with an opportunity to design and implement quality family medicine and to increase the quality in family medicine. Although it has assumed this central role feature well so far, Singapore may need to be supported politically and economically. To update, change and raise the standards of family medicine and reach a global example, quality-focused countries such as Singapore should be evaluated from the perspective of the Göktaş Definition of Family Medicine/General Practice.
